# Directional Collective Cell Migration Emerges as a Property of Cell Interactions

**DOI:** 10.1371/journal.pone.0104969

**Published:** 2014-09-02

**Authors:** Mae L. Woods, Carlos Carmona-Fontaine, Chris P. Barnes, Iain D. Couzin, Roberto Mayor, Karen M. Page

**Affiliations:** 1 Centre for Mathematics and Physics in the Life Sciences and Experimental Biology, University College London, London, United Kingdom; 2 Department of Cell and Developmental Biology, University College London, London, United Kingdom; 3 Department of Ecology and Evolutionary Biology, Princeton University, Princeton, New Jersey, United States of America; 4 Department of Mathematics, University College London, London, United Kingdom; Queensland University of Technology, Australia

## Abstract

Collective cell migration is a fundamental process, occurring during embryogenesis and cancer metastasis. Neural crest cells exhibit such coordinated migration, where aberrant motion can lead to fatality or dysfunction of the embryo. Migration involves at least two complementary mechanisms: contact inhibition of locomotion (a repulsive interaction corresponding to a directional change of migration upon contact with a reciprocating cell), and co-attraction (a mutual chemoattraction mechanism). Here, we develop and employ a parameterized discrete element model of neural crest cells, to investigate how these mechanisms contribute to long-range directional migration during development. Motion is characterized using a coherence parameter and the time taken to reach, collectively, a target location. The simulated cell group is shown to switch from a diffusive to a persistent state as the response-rate to co-attraction is increased. Furthermore, the model predicts that when co-attraction is inhibited, neural crest cells can migrate into restrictive regions. Indeed, inhibition of co-attraction *in vivo* and *in vitro* leads to cell invasion into restrictive areas, confirming the prediction of the model. This suggests that the interplay between the complementary mechanisms may contribute to guidance of the neural crest. We conclude that directional migration is a system property and does not require action of external chemoattractants.

## Introduction

The Neural Crest (NC) is a multi-potent cell population that arises at the dorsal midline during embryo development, migrates ventrally through the embryo and is guided by strict migratory pathways [1]. Collective cell migration is an important biological process that occurs during development [2], wound healing [3], cell renewal [4]–[6] and metastasis [7]. Recent efforts have identified the NC as a suitable model for collective cell migration [8], [9] and for metastasis, as similarities between the NC and metastatic cancer cells have been observed [10], [11]. The mechanisms that regulate collective cell migration are not fully understood, however data suggests cranial NC cell migration both *in vivo* and *in vitro*, is regulated by 1) contact inhibition of locomotion (CIL) [12], [13], 2) chemotaxis towards a self secreted chemoattractant [14] and 3) a collection of external negative signalling molecules such as Eph/Ephrin and Robo/Slit, for a review see [8]. CIL was discovered by Abercrombie and Heaysman [15], [16] and has been extensively studied in a range of experimental systems [17]–[21]. Mechanically, CIL can be described as a change in motion of individual cells due to contact, and occurs to differing extents in migratory cell types, such as fibroblasts [15]–[17], keratinocytes [22], *Drosophila* macrophages [23], NC [12], [18]–[21] and the PC-3 cancer cell line [16], [22]–[24]. This process has been characterized in *Xenopus*, chick and zebrafish NC and has been demonstrated as a key mechanism that confers cell polarity, by regulating the activity of small GTPases, and controlling directional migration of the whole NC population [12], [25]

Attraction between NC cells has been observed to take place concurrently with CIL [14]. The complement factor C3a and its receptor C3aR were found expressed in the migrating NC and previous work has demonstrated that C3a plays a direct role in collective migration, functioning as a homogenous NC secreted chemo-attractant. This chemokine acts to maintain a high cell density through homotypic attraction, a phenomenon called co-attraction [14]. As CIL and co-attraction have been described as two microscopic processes with opposite effects on NC cells (repulsion and attraction, respectively), it is not evident how the relative contribution of these two contrary forces could affect directional migration of a group of cells. To better understand whether CIL and co-attraction could control directional migration of NC cells we developed a mathematical model of the process.

In previous studies, agent based models have addressed the transition from disordered to ordered motion in swarming insects [26] and the dynamics of wound healing assays [27]. These models assign rules to individual agents, from which, changes in local interactions lead to phase transitions, such as a parallel to a torus state [28]. Collective migration in real cells has been compared to flocking behavior modeled in the coordinated movement of animal groups and experiments have confirmed shared properties such as local correlation and responses to the local environment [29], [30]. Further analysis in agent based methods has led to analytic approximations on the force required to maintain a particular state [31] and quantification of adaptable interactions to the local environment [32]. Force-based models have provided an alternative descriptive modeling approach allowing parameter prediction based on a macroscopic feature such as group alignment. In a study on keratinocytes, long-range order was shown to depend on repulsive and adhesive forces [33] and in a mechanical model, wave propagation has been described in epithelial monolayers [34].

Several models have been proposed [35]–[37] in which NC cells have been assumed to migrate in response to external chemoattractant gradients. Although there is evidence to support the presence of NC chemoattractants *in vivo* [38]–[40], it has been very well documented that NC cultured *in vitro* in the absence of any external chemoattractant exhibit directional collective migration [40], [41]. The effect of random perturbations in collective migration has been analysed [35] and the stability of NC chains characterized [37]. In the study of Wynn *et al.*, agents were simulated on a grid and parameter analysis was performed on an initial pattern to test chain persistence with leader and follower cells. Further investigation suggested that cell interactions with the ECM, directional bias and cell contact could play a mutual role in the promotion of chain migration [42]. A different study that compared theory and experiment used an off-lattice individual based model combined with a continuous model of vascular endothelial growth factor to predict behaviour of cranial NC migration in chick. The results of this study suggested that a combination of leading and trailing cells are required to ensure cohesive movement and collective response to external signals [36]. Alternative models have demonstrated network formation in the absence of external gradients, for example where simulated cells are cued by strains [43] and in the NC, where rules of movement include agent path reinforcement and repulsion or preference to follow existing axons [44].

In this work, a model of NC collective migration is presented. In a similar fashion to a study on fibroblast migration [45], microscopic parameters are estimated from biological data analysis and through simulation, macroscopic features of migration are predicted and compared with experimental data. In contrast to Vedel *et al.* [45], where the effect of local parameters on relative simulated cell dynamics was assessed in the form of an autocorrelation function, we focus on both correlations between velocities and collective properties of the group in the form of a time taken to reach, collectively, a target location, allowing us to assess long-range dynamics. When a group of NC cells is plated on fibronectin, they are able to migrate collectively, and with directionality, in the absence of any external signal. Additionally when the leading edge is removed, previously trailing cells continue migrating assuming a leading phenotype [12], [14], [40]. Previous models have investigated leading and trailing populations [46] but some have employed different mechanistic rules amongst simulated cells [37]. Models that include predefined differences between leading and trailing cells do not take into account the emergent heterogeneity that can arise as a consequence of the dynamical system. In addition, it has previously been shown that a combination of repulsive (CIL) and attractive (co-attraction) forces could generate directional migration [14]; however, this model did not consider the migration parameters of real cells. Although the model can generate an efficient migration when CIL and co-attraction are combined, it does not reproduce the real behaviour of cell clusters when only CIL is present or the real behaviour of single cells. Hence, there is a need to construct a model that reflects the biological observations in the cranial NC that will be better suited for comparison with functional experiments.

## Methods

### The model

**Figure 1:**
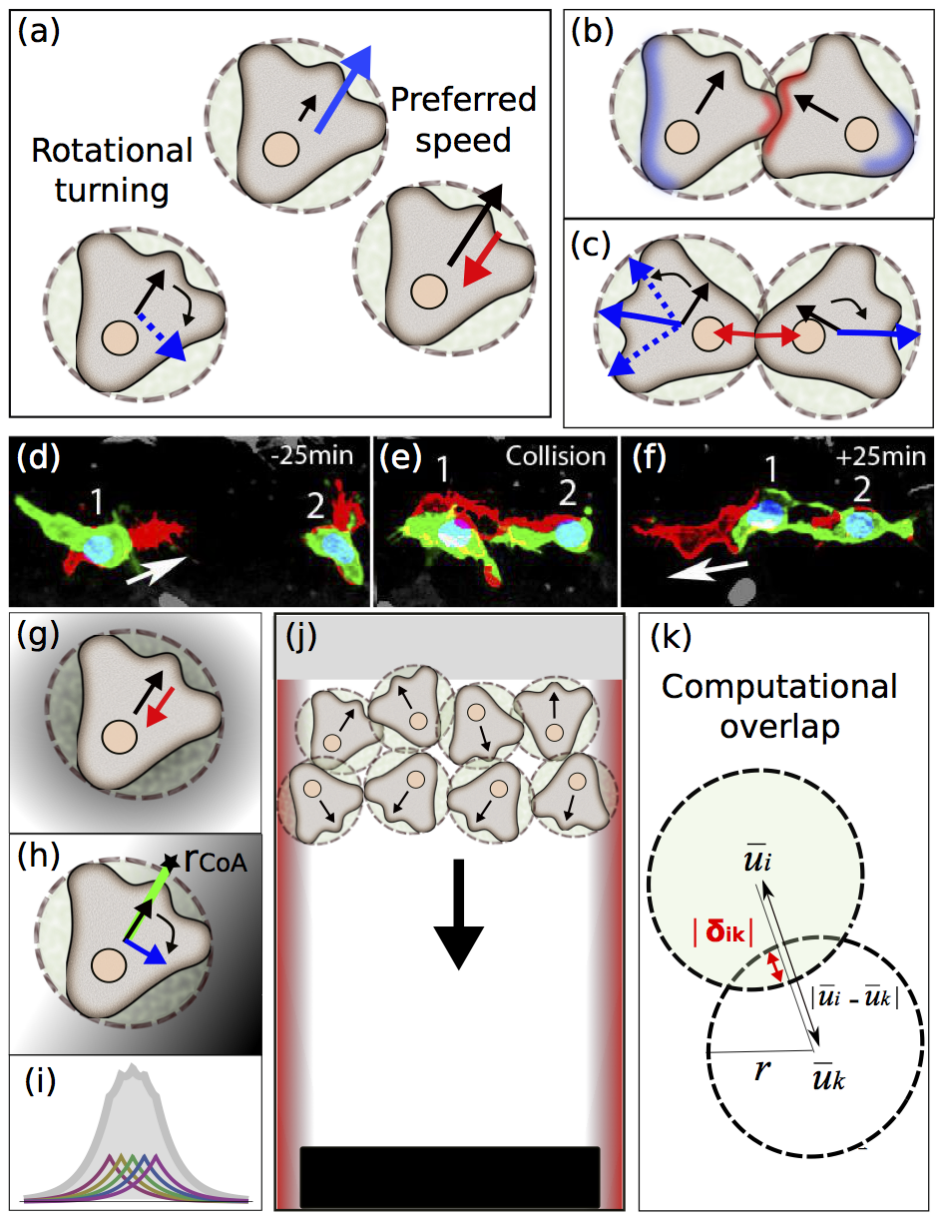
Discrete element model. Disks (broken lines) represent simulated cells, with cartoon NC cells overlaid. The polarity of each cell is shown (black arrow) and the forces attenuating or amplifying protrusion formation are indicated with red and blue arrows (respectively). (a). Self-propulsion and rotational turning (blue dashed arrow) force terms: cells attempt to maintain an intrinsic speed and polarity by acceleration (blue arrow), and deceleration (red arrow). (b). CIL: as cells come into contact, contact forces exist at the contact region. In addition, biological intracellular communication promotes the retraction of protrusions near the contact site (red region). Intracellular communication affected by contact promotes protrusion formation at the free edge (blue region). (c). Forces applied during CIL: classical overlap and repolarization are indicated (solid red and blue arrows). Deviation from the classical theory of contact is represented as a random angle (blue dotted arrows). (d–f). Frames of a time-lapse movie of zebrafish NC migrating in vivo. Green labels GFP expressed in the NC under Sox10 regulatory elements; Red: cell protrusions. Two cells (1 and 2) are shown. Arrow: direction of migration for cell 1. (d). Before contact. (e). During contact protrusions collapse. (f). After CIL, protrusions are generated at the free edge. (g). Secretion of co-attractant: at the single cell level the cell experiences a retraction of protrusion. (h). Co-attraction for multiple cells: individual cells experience the co-attractant generated by the whole population and attempt to align their polarity to the gradient with the sensing ray (green line, star indicates the location of the measurement). (i). Typical surface profile of the co-attractant: individual sources and their sum are shown (colour and grey plots resp.). (j). Simulation set up, designed to represent NC streams *in vivo*: Cell positions represent the origination and initial conditions of the NC. The vertical borders represent the restrictive cues that surround the migratory stream. (k). Diagram representing the computational overlap, where overlap represents the deformation of the cells.

We present a microscopic model based on a periodic change in polarity, resulting in a change of direction, which we call rotational turning (Figure 1a) and the processes CIL and co-attraction. Measured properties of these interactions (see Text S1, Figure S1), taken from *Xenopus* NC cells migrating *in vivo* and *in vitro*, are incorporated to the model, which follows the discrete element method [47].

We abstract NC cells to elastic spheres that we refer to as simulated cells. For a population of size 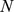, each simulated cell is equipped with a natural radius 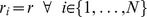 and a ray 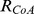 in the direction of polarity corresponding to the sensing range of the simulated cell. In addition, each simulated cell is assigned a mass 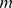 and intrinsic speed 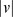. In the event that contact occurs between simulated cells, normal contact forces are modelled with Hertz contact theory (see Text S1). Data analysis of CIL *in vitro* confirms that the mechanism of contact inhibition is significantly different from the dynamics of an equal mass normal force rigid body collision. To account for this, the model is modified through the addition of a repolarisation force that acts in a randomly distributed direction at the free edge, see (Figure 1b–c). This implementation is different to previous models of swarming that have assumed inelastic collisions [48] and is consistent with experimental data, as the generation of protrusions at the free edge has not only been observed *in vitro* in Xenopus but also *in vivo* in Zebrafish, see (Figure 1d–f). Single NC cells observed *in vivo* periodically change their direction of migration [12], [49]. This change in direction of migration is dependent on the direction of their protrusions and can be observed by plotting individual cell tracks or recording cell persistence. To account for this behavior in our model, each simulated cell is assigned two internal clocks that periodically switch on a force due to co-attraction and an impulsive force due to rotational turning. Currently these rates are unidentified experimentally. In the event that a simulated cell responds to co-attraction, the simulated cell is subjected to a force proportional to the gradient of the co-attraction profile, as the steepness of external gradients have been shown previously to affect cell motility in eukaryotic cells [50] (Figure 1g–i). Simulations were performed in a 2D continuous geometry, to represent the permissive extra cellular matrix, with a rigid wall at the dorsal border and a repulsive cue at the lateral borders to represent negative signals that are known to be present in the embryo at the border of each NC stream. It is known that some of these molecules are secreted, like semaphorin [8], which would generate a gradient consistent with the model. In the event that a simulated cell responds to the lateral repulsive gradient, it is subjected to a force proportional to the gradient, which is localised at the border. The domain is equipped with a target at the opposite end of the domain from the initial location of the simulated cells see (Figure 1j). When a simulated cell reaches the target, it remains stationary for the remainder of the simulation, which represents the real cells ceasing migrating once they reach the target tissue in the branchial arches. The extraction of the simulated cells facilitates the analysis of efficiency in directional migration by quantifying the number of cells that reach the target (see Text S1).

### Time integration

The dynamics of cellular motion are driven by the sum of the applied forces. Simulated cells maintain a ‘preferred’ self-propelled velocity 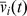 in the absence of the forces tested. This is an abstraction of the real biological scenario, where the velocity of migration is generated mainly by actin polymerization at the cell front [51] and single cells exhibit differences in their velocity over time. However this simplification allows us to more clearly explore how interaction forces influence the group level dynamics. A simulated cell always moves in the direction of its polarity. The force that governs the migration of a simulated cell is presented below and simulations were performed with the iterative central difference model [47].
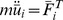
(1)

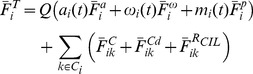
(2)Here, 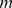 is the mass, 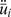 is the acceleration of the simulated cell with position vector 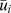. 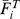 is the total interaction traction force that will influence a change in the velocity. 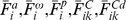 and 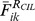 are the co-attraction, rotational turning, self-propulsion, contact, contact damping and contact repolarisation forces (see Text S1). 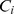 is the set of indices of simulated cells in contact with simulated cell 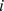 (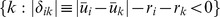, see Figure 1k). The coefficient 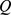 sets the co-attraction, self-propelled and rotational turning forces to zero when a simulated cell is in contact and the coefficients 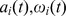 and 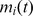 are functions of the internal clocks for co-attraction 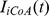 and rotational turning 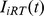 (see Text S1).

### Model Calibration

Where possible we have attempted to match model parameters to the control real cell biological data. Following Wynn *et al.* and Carmona-Fontaine *et al.* [14], [37] baseline parameters were chosen that correspond to physiological conditions and are presented in Table S1. The computational domain was defined with a height of 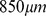 and width of 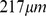. The simulated cell diameter was uniformly defined as 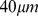 to approximate the cell width observed *in vitro* and *in vivo*, with the simulated cell speed estimated from biological data as 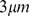 every minute (5.0e-8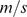) [12].

**Figure 2:**
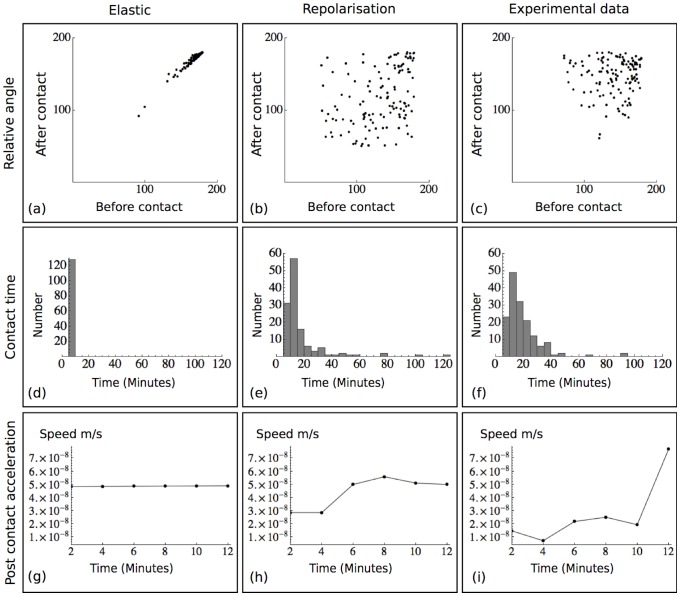
Calibration to *in vitro* data. (a). Relative angle of the normal force elastic collision model, representing equal mass impulse momentum. (b). Relative angle of the repolarization model. (c). Relative angle of the biological data. (d). Contact time for the normal force elastic collision model. (e). Contact time for the repolarization model. (f). Contact time for the real cell data. (g). Mean speed after contact separation of the normal force elastic collision model. (h). Mean speed after contact separation for the repolarization model. Note that the speed does not increase past the default migratory speed due to the self-propulsion force term. (i). Mean speed after contact separation for the real cell data, average over 4 cells.

To construct a model that represents the microscopic interaction of the real cells during contact inhibition, we analysed three quantitative values to parameterise force equations based on the theory of contact mechanics. These values were the angle before and after CIL, the contact time and the acceleration after contact. During contact and CIL, cellular motion is modelled as a function of the normal contact force and a repolarisation force. These two forces represent the material properties of a cell and the fact that CIL activates a molecular signalling pathway, which affects molecular activity at the free edge, promoting protrusion formation. It is known that protrusions are inhibited at the site of contact, via a mechanism involving cadherins and Rho-GTPases [52]–[54] (for a review see [55]). In addition, the PCP pathway regulates repolarisation at the free edge [12]. The repolarisation force is not present in standard discrete element models. The force acts in the direction of the unit vector connecting the two cells centre's of mass plus a random angle sampled from a uniform distribution with the range 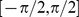 (see Text S1). The exact distribution does not have a significant effect on the contact model, see Figure S2a–b. To test the influence of the normal contact force on the collective dynamics of the cells and to understand whether the relative velocity of two cells during contact was a significant factor in the model, the angles between the paired velocities of two biological cells prior to and following contact were analysed by assessing if they were correlated. Where possible microscopic parameters were approximated with real cell data from the literature [12], [14], [56]. The simplest form of contact is that of a normal force rigid body elastic collision, which we expected would give rise to highly correlated pre- and post- contact angles. First we tested whether these angles were independent in the experimental data. By assuming that the pre- and post- contact angles have a bivariate normal distribution, testing for independence becomes equivalent to testing whether the correlation coefficient 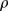 is zero. The hypothesis 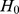 that the pre- and post-contact angles exhibited a correlation coefficient 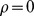 was tested for the whole data set using a two tailed t test with test statistic 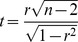, where 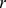 is the sample correlation coefficient and 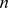 is the sample size. The biological data yielded a sample correlation coefficient of 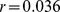 and a statistic 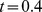 for 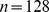, which suggests that the pre- and post-collision angles are not correlated (Figure 2c). This test was repeated for cells that remained together for at least the mean time yielding 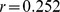 and 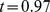, which again supports the hypothesis that pre-collision angles are not correlated with post collision angles. A low correlation indicates that the predominant forces involved in contact inhibition are not due to normal contact forces but some other mechanism, which we define loosely as repolarisation. To compare these results to the model, a normal force elastic body collision scenario was tested. This model neglects the terms 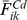 and 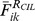, which constitute the energy dissipation and repolarisation forces. The normal force model alone in the absence of these terms exhibited an r value of 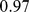 and a test statistic 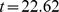, indicating that pre- collision and post-collision angles are correlated (Figure 2a). When repolarisation and contact damping were included, the model exhibited an r value of 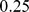 and 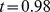, indicating that the pre- and post-collision angles are not correlated as in the case of the biological data, see (Figure 2b). We then looked at the time that cells remain in contact. Experimental movies had a frame rate of 5 minutes so that the minimum contact time recorded would be less than 10 minutes. The experimental distribution in time was compared to both the normal force elastic body collisions and the repolarisation model, (Figure 2d–f). During CIL and contact separation, it takes some time for the real cells to regain their default migratory speed. There is variation in this acceleration. To obtain quantitative data on this process, the speed of a real cell upon contact separation was recorded over time. The results confirm that to regain the default speed, cells must accelerate after contact, which suggests that CIL cannot be fully described by a normal force rigid body elastic collision. Speed after contact was recorded for the repolarisation model. In contrast to the experimental data, the simulated cell was unable to accelerate to 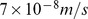, however this is due to the default speed being set to 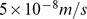. When the default speed was increased to 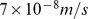, the simulated cell's speed increased to a value greater than 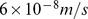, see Figure S2c. Compared with the normal force elastic model, the repolarisation model can better explain the change in speed after contact, (Figure 2g–i). Together these results suggest that rigid body collisions are not sufficient to model contacts between cells, but that our model, which incorporates a novel repolarization force can better do so.

The frequency of the clocks cannot simply be measured. Therefore conclusions on the effect of these parameters were drawn after parameter analysis. We take a baseline rate for chemotactic response to be one response every two seconds and for reorientation, one random change in direction every five minutes.

We have shown that C3a (the co-attractant) forms a stable gradient by binding to fibronectin [14]; this gradient was measured and a 2D mathematical radial diffusion model was assumed. To model the chemoattractant, we assume a steady state distribution at every iteration, as the timescale for diffusion is smaller than the time it takes for a cell to move a significant distance. This steady state distribution can be described as a Bessel function [57], which for simplicity we approximated with a decaying exponential with a half maximum length 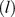 of 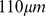.

## Results

### Co-attraction and CIL are sufficient and necessary (*in silico*) for directional collective migration

**Figure 3:**
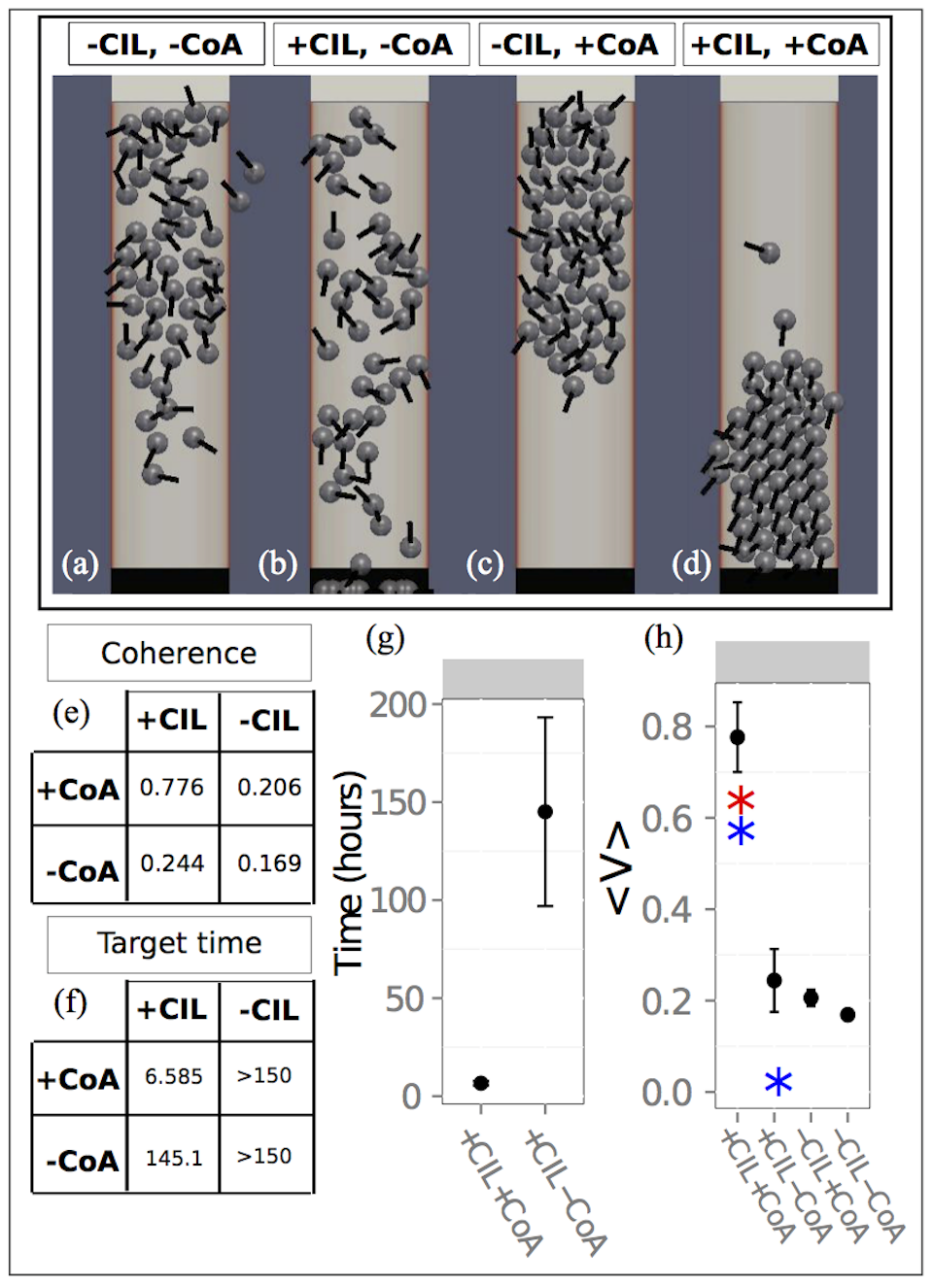
Relationship between CIL, co-attraction and collective migration. (a–d). Images taken at approximately half of the baseline collective target time. (a). **−CIL,−CoA**, where migration can be seen to be less efficient than in (b) and (d). (b). **+CIL,−CoA** (c). **−CIL,+CoA**, (d). **+CIL,+CoA**. (e). Table of coherence measures for the four cases. (f). Table of collective target times for the four mutually exclusive cases. (g). Collective target time for the cases **+CIL,+CoA** and **+CIL,−CoA**. The cases **−CIL,−CoA** and **−CIL,+CoA** are omitted as the time was greater than 150 hours. (h). Coherence measure of the four cases, shown in black. Blue star indicates the automated tracking value for the model and the red star shows the experimental data tracking software value.

Migration is found to be a qualitative fit to the behaviour observed for real cells [14], for example, in the absence of an external bias the cells migrate in a coordinated fashion leading to the displacement of the group as a whole. This suggests that the model can reproduce directional migration with the functional processes CIL, co-attraction and rotational turning. In the presence of CIL and co-attraction, directional migration occurred as a travelling wave of density, which reproduces the directional migration observed in real cells [12], [14]. To test the relationship between directional collective migration, co-attraction and CIL, four cases were considered: (1) **−CIL,−CoA** corresponding to an elimination of all processes except rotational turning, (2) **+CIL,−CoA** representing a complete knockdown of co-attraction, (3) **−CIL,+CoA** which tests the model under the assumption that CIL is inhibited and (4) **+CIL,+CoA** corresponding to the baseline case (Figure 3a–d, Video S1). Out of all four cases **+CIL,+CoA** produced the most efficient migration through the domain, in which the centre of mass of the group was the most distal at a simulation time of approximately 2 hours. To quantify this efficiency, we define directional migration as the combination of a high coherence and low target time (Text S1), where high coherence corresponds to a value greater than 0.5. Case (4) was unique in displaying these properties, suggesting that both CIL and co-attraction are necessary for directional migration in the model (Figure 3e–h).

In cases where co-attraction is absent, the macroscopic behaviour is similar to diffusion in a bounded domain (Figure 3a–b). To assess if directional migration requires CIL, the model was simulated in the absence of 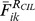 and with continual 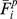 during contact. In contrast to the dynamics of a co-attraction knockdown, elimination of CIL resulted in minimal displacement of the bulk population (Figure 3c). These results were upheld under further analysis of velocity, where coherence was high under baseline conditions and low when either CIL or co-attraction was impaired (Figure 3e–h). To compare the model predictions with biological data, automated tracking software was used on both the model and experimental data at the same frame rate frequency [58]. Individual simulated cell and cell velocity was tracked and the coherence computed for a control experiment, the baseline parameters and a knockdown of co-attraction in the model. Under baseline conditions, the software calculated a coherence of 0.6252. To analyse the predictive quality of the model, control experimental data was processed and exhibited a coherence of 0.5568. To test the goodness of fit of these values, a simulation lacking co-attraction was processed. For this case, the coherence was 0.008, suggesting that baseline parameters are a better fit to control migration and they reproduce the cell behaviour observed *in vivo* and *in vitro* (see Figure 3h, Figure S3a–c).

### Migration is diffusive if co-attraction is not sufficiently strong

**Figure 4:**
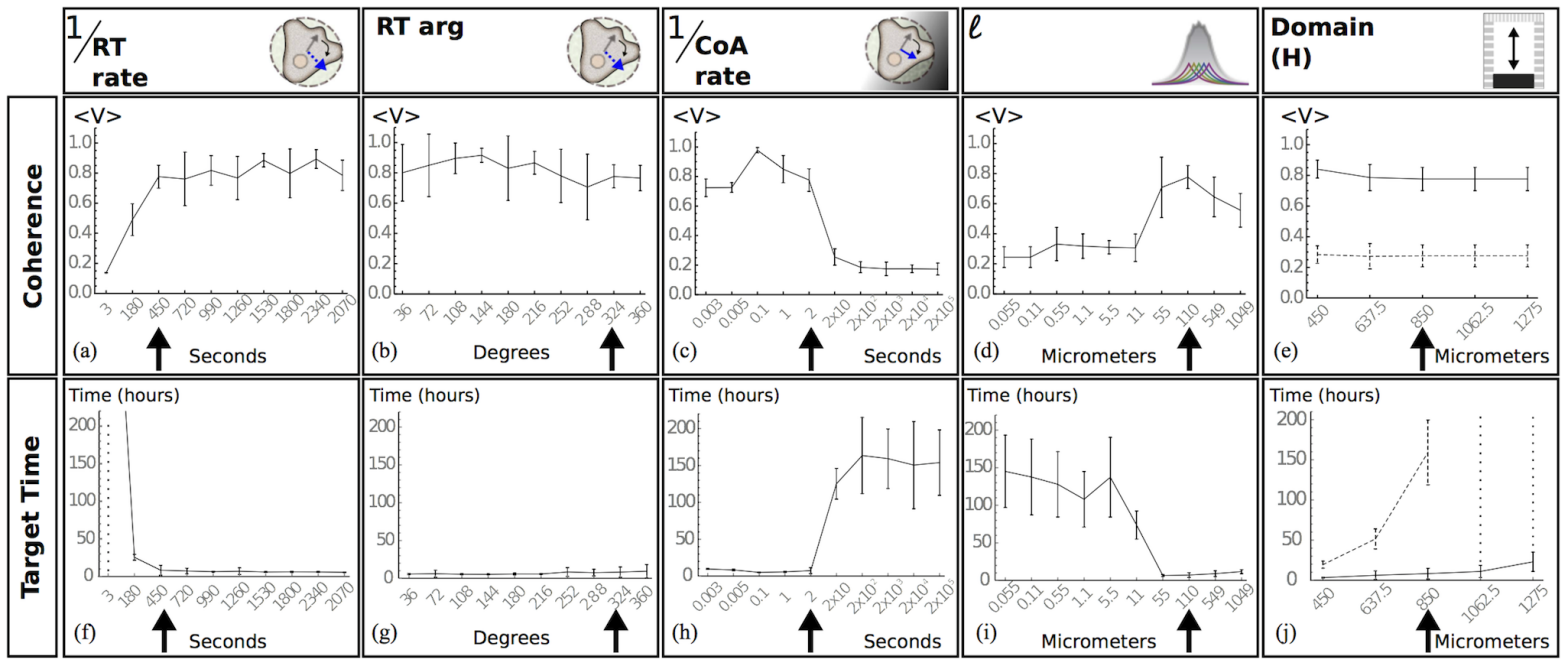
Coherence measure and collective target time. Data points represent the mean value over 10 independent simulations and error bars represent one standard deviation from the mean. Black arrows represent baseline parameter values. (a). Variation of 1/(RT rate) showing coherence. (b). Variation of the angle by which the cells can deviate during RT showing coherence. (c). Variation of 1/(CoA rate) showing coherence. (d). Variation of the diffusion length showing coherence. (e). Variation of the domain width showing coherence for weak (dashed) and strong co-attraction (solid line). (f). Variation of 1/(RT rate) showing collective target time. (g). Variation of the angle by which the cells can deviate during RT showing collective target time. (h). Variation of 1/(CoA rate) showing the collective target time. (i). Variation of the diffusion length, showing the collective target time. (j). Variation of the domain width showing the collective target time for weak (dashed) and strong co-attraction (solid line).

**Figure 5:**
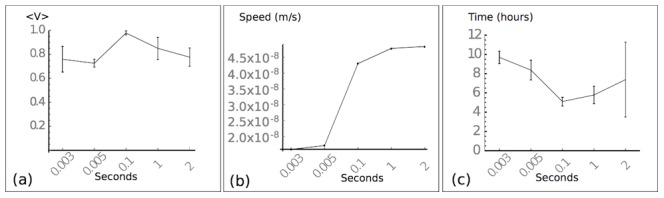
Optimal rate of response to co-attraction. System behaviour for strong co-attraction, showing an optimal rate at 1/(CoA rate)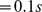. (a). Coherence is maintained for high rates of co-attraction. (b). Speed is reduced as the rate of co-attraction increases. (c). Collective target time. The smallest target time occurs at 1/(CoA rate)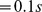.

To test the robustness of directional migration sensitivity analysis was performed by considering the effect of one parameter at a time on directional migration. This was implemented for five physiological parameters, consisting of the C3a diffusion length 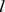, the angle by which the simulated cells can deviate during rotational turning, the rates of the internal clocks 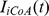, 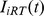 and the domain length (Figure 4). Collective migration occurred in baseline simulations and was maintained under small parameter variation. There was variation in the collective target time for baseline parameters between independent simulations. This variation is negligible when compared to the collective target time for the diffusive state and we refer to the time in which the group remains travelling in one direction as the collective flight time of the group (see Figure S3d–f, Video S2a). We performed a Mann-Whitney U test on the coherence between consecutive parameter values presented in Figure 4a–e to test for a difference in medians between consecutive parameter values. In agreement with previous studies, frequent reorientation resulted in a low coherence (1/(RT rate)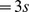, Figure 4a,f) and there is evidence to suggest that there is a difference in the median coherence between parameter values 1/(RT rate) 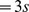 and 1/(RT rate) 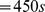, (p<0.0005, n = 10). For baseline parameters, different angles by which the simulated cells can deviate did not disrupt collective migration (Figure 4b,g) and there was no significant difference in the median coherence between consecutive parameter values, (p>0.01, n = 10). The effect of co-attraction on group level dynamics was tested by variation of 1/(CoA rate) and the diffusion length 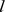 (Figure 4c,d,h,i). The results show that simulated cells fail to directionally migrate when there is an infrequent response to co-attraction, or if the co-attraction gradient is too short-range. For example, there is a significant difference in the median coherence between a response parameter of 1/(CoA rate)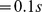 and 1/(CoA rate)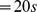, (p<0.0005, n = 10). Similarly for the gradient, there is a significant difference in the median coherence between 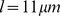 and 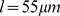 (p<0.005, n = 10). This co-attraction dependent transition between directional migration and dispersion occurred at a spatial occupancy of 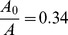, where 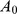 is the area occupied by the cells and 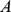 is the total area and this density occupancy was held constant across all simulations. In previous studies of epithelial cell populations, group coherence is exhibited with densities greater than 0.2 [33]. This result suggests that mesenchymal cell populations such as the NC, may naturally disperse in the absence of a co-attractant but the response to co-attraction regulates this behaviour and allows cells to acquire motion similar to those of epithelial cell types. To characterise the transition from diffusive to directional collective migration, coherence and target times were recorded under variation of the box height H, for weak and strong co-attraction (Figure 4e,j). The coherence was high and the collective target time increased linearly with H for strong co-attraction. In contrast the coherence was low and the target time increased super linearly for weak co-attraction (Figure 4e,j). To obtain an upper bound on the rate of co-attraction, we considered small values of 1/(CoA rate). The coherence was recorded for values between 1/(CoA rate)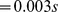 and 1/(CoA rate)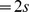, (Figure 5a). Coherence was maintained within this range. In contrast to this, the speed of the simulated cells changed, such that the average speed of a simulated cell during 1/(CoA rate) 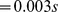 is 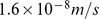. This suggests that although coherence is maintained at high rates of co-attraction, efficiency of bulk displacement is reduced. Furthermore, a reduction in speed coincides with a longer collective target time, suggesting that there is an optimal response rate to co-attraction. The collective coherence, speed and collective target time were recorded for five different rates of 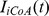 (Figure 5a–c). From this data, we suggest that although there exists a range of 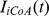 rates within which directional migration can emerge (see error boundaries, Table S1), the optimal value for the parameters tested coincides with 1/(CoA rate)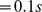.

### Model predictions

**Figure 6:**
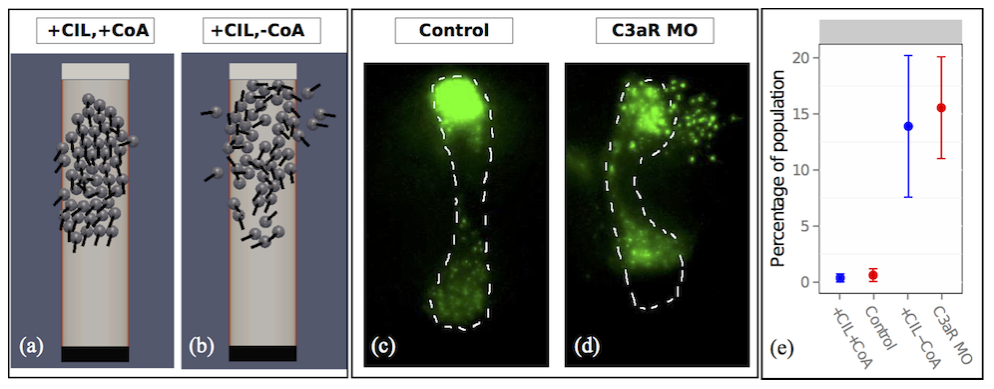
Co-attraction facilitates stream guidance. (a). **+CIL,+CoA**, cells respond to the restrictive cues and remain in the migratory region. (b). **+CIL,−CoA**, cells neglect restrictive cues and migrate into the restricted region. (c). Control NC cells migrating *in vivo*. (d). C3aR MO cells migrating *in vivo*. In this case single cells cross into restricted regions. (e). Quantification on the percentage of the population that has moved into the restricted region throughout simulation (blue) and *in vitro* experiment (red).

Under baseline conditions, simulated cells remained positioned within the permissive regions during migration to the target (Figure 6a). In contrast, when co-attraction was inhibited, we noticed that single simulated cells appear to cross into the lateral restricted regions (Figure 6b). To quantitatively validate this behaviour, we recorded the average number of simulated cells that reside in the restricted region for a response to the boundary signal every 9 s. This number was recorded as a percentage of the population for cases (2) and (4) in the model. On average, the percentage of cells that crossed the lateral border was close to 0% in the baseline condition, whereas it was close to 14% when co-attraction was inhibited (blue in Figure 6e). To test whether this unexpected prediction of the model was also found in real cells, an experiment to reduce co-attraction *in vivo* was performed. An antisense morpholino was used against C3aR to inhibit co-attraction. In this experiment, control cells remain positioned within their migratory streams, (Figure 6c) however, invasion of cells into non-permissive area was observed for cells depleted of C3aR (Figure 6d). To quantitatively compare simulated and real cells, an *in vitro* experiment was performed. NC explants were cultured on corridors of fibronectin flanked by fibronectin-free regions, and time lapse analysis was performed, (see Figure S4). NC cells need fibronectin for their migration as they inefficiently attach to a fibronectin-free substrate. While control cells rarely invaded the fibronectin-free region (Figure S4a) an important proportion of the C3aR depleted cells moved into that region (Figure S4b). The percentage of cells invading the prohibited region was similar between the model and the real cells (Figure 6e). The average percentage of cells to cross into the restricted region throughout the simulation was recorded for different boundary clocks 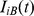 (see Text S1). For values of 1/(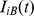 rate)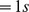, 1/(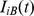 rate)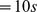 and 1/(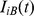 rate)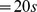, the average percentage of cells to cross into the restricted region for control simulated cells remained within 5%. For 1/(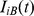 rate)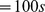, the average percentage of cells to cross into the restricted region for control simulated cells was greater than 50%. The model predicts that co-attraction facilitates guidance of the NC by counteracting the migratory force that is sufficient to overcome negative signals. By assigning a force term to the processes of co-attraction in the model, we were able to compare model predictions with functional experiments. For this behaviour, the results of the model and experiment are in agreement.

Emergent behaviour was not limited to stream guidance. Previously, NC explant confrontation was performed to directly test co-attraction. In this experiment, explants are cultured within a distance that is great enough to ensure that no initial contact occurs between the groups. It is known that the groups consistently move toward each other, however the number of cells in each group determines the distance over which co-attraction can act. This property was consistent with the model, where groups of NC cells respond to co-attraction at greater distances than single cells (see Video S3, S4, Figure S5). Differences in velocity were observed between leading and trailing cells when co-attraction occurred at every 0.1 s or less (see Figure S6, Video S2b). Leading and trailing behaviour has previously been shown to occur in the chick and *Xenopus* NC [12], [37], [40]. We suggest that this could emerge in a population of identical cells without requiring differentiation of microscopic parameters, as it has been shown for NC migrating *in vitro* [12].

## Discussion

In this study, two processes (CIL and co-attraction) that occur in migratory cells are analysed with an application of the discrete element method. This method is commonly applied in the field of mechanical engineering and here we apply it to cell migration.

Analysis of experimental data on cell collisions shows that normal contact forces alone cannot well describe these collisions. The inclusion of a repolarisation force, generated by the retraction of protrusions at the contact site and the formation of new protrusions at the free edge, allows a much better fit to data.

Coherence and efficiency of bulk displacement of simulated cells indicate that CIL and co-attraction are both sufficient, and necessary, for effective directional migration. Upon inhibition of either process, collective migration is disrupted and the dynamics consist of high cell density meandering clusters in the absence of CIL or low cell density random movement in the absence of co-attraction. Qualitative and quantitative measurements of the global dynamics of the simulation are compared with experimental data and application of automated tracking software [58]. We identify a range of possible values for the co-attraction rate. By designing a model parameterised with experimental data at the microscopic scale, we demonstrate that directional migration is robust to small changes in the processes of CIL and co-attraction, however both infrequent and continual co-attraction can disrupt timely directional migration. This model allows us to explore the effect of variation in the microscopic parameters on collective behaviour, to support existing experiments or to make predictions when real experimental values are unknown.

In contrast to previous studies, our model predicts that co-attraction contributes to the guidance of the NC by promoting directional migration and inhibiting single cells from migrating into restricted regions. Here, we provide experimental evidence that confirms the model prediction *in vivo* and *in vitro*.

Where feasible, processes are parameterised using experimental data. This uncovers a timescale suitable for modelling contact, a dynamical process with resolution on the millisecond scale. To assess and quantify long-range dynamics, simulations are on the scale of hours. To facilitate these intensive simulations, the code is implemented in CUDA (Compute Unified Device Architecture). This allows us to exploit the highly parallel nature of graphical processing units (GPUs) for quantitative analysis.

Previous models, have shed light on the emergent dynamics arising from individual interactions inspired by biological data, however several have depended on the presence of an external chemo-attractant [35]–[37]. By contrast, this study does not invoke an external chemoattractant but considers instead chemotaxis towards a self-secreted chemoattractant C3a and tests the role of co-attraction in collective migration and stream guidance. Biological evidence suggests that individual interactions between cells work together to allow self-organization in migrating clusters and collective migration [12], [14]. This study confirms this from a mechanical perspective and suggests that CIL and co-attraction promote migration similar to epithelial directional migration in cell populations that are mesenchymal with low cell-cell adhesion. In addition to CIL and co-attraction, external signalling, such as chemoattractants and chemorepellents have been shown to play a role in NC migration [1]. The model presented here suggests that long-range directional migration is acquired through local tissue specific interactions and permissive cues that have the greatest effect on migrating collectives. This could potentially promote a flexible system that is ready to adapt to external and internal perturbations.

## Supporting Information

Figure S1 **Contact time.** (a). Frame from an *in vitro* experiment showing the contact area of two cells. (b). Frame after 10 seconds have elapsed from the time of frame in (a). (c). Length of contact area cross section, recorded over 10 seconds, with data from the experiment shown in (a) and (b).
(TIFF)

Figure S2
**Testing repolarisation.** (a–b). Repolarisation plots. (a). Exponential distribution with mean 18 degrees from normal vector connecting the cell's centre of mass. (b). Uniform distribution between 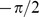 and 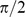. (c) Speed after contact for the parameter value 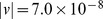.(TIFF)

Figure S3 **Tracking cell motion.** (a). Time frame from the DIDSON tracking software for the model case (**+CIL,+CoA**). (b). Time frame from the DIDSON tracking software for a control group of NC cells plated on a strip of fibronectin. (c). Time frame from the DIDSON tracking software for the model case (**+CIL−CoA**). (d). Directional migration for the baseline case, showing the cosine of the angle made with the vertical axis for the average group direction. Average distribution of direction over the whole simulation, 10 independent simulations shown in different colours. (e). Time series of a single simulation, showing that persistence of direction can last for up to an hour in length and switches in direction can take place in a few minutes. (f). Time series for a single simulation, showing that a group can continually move in one direction for two hours subject to a few reorientations.
(TIFF)

Figure S4 **Co-attraction facilitates stream guidance.** NC cultured on corridor of fibronectin (black area), flanked by non-permissive substrate (red area). (a) Control NC. (b) C3aR deficient NC, here cells are able to cross into the restricted region.
(TIFF)

Figure S5
**Co-attraction between two different sized groups.** (a). 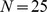, 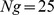, initial condition, where the centre of mass separation was 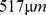. (b). At a time of 51 minutes into the simulation, the groups begin the join. (c). At a time of 210 minutes, the two groups have responded to co-attraction and collectively migrate in a random direction. (d). Initial condition for the case 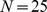, 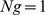. (e). At time 51, in contrast to the simulation shown in (*B*), the single cell is disjoint from the larger group. (f). Time series showing the centre of mass separation for the three distances analysed, (see initial condition at time zero). At a distance of 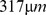 the single cell can migrate towards the reference group. (g). Same plot as shown in (f), for the condition 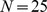, 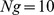. (h). Same plot as shown in (f), for the condition 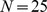, 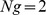. (i). Same plot as shown in (f), for the condition 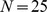, 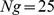. (j). Table showing the results of the model and experiment. Time at which the groups have joined and the threshold at which groups can respond to co-attraction for all cases analysed.(TIFF)

Figure S6 **Leading and trailing cells.** Images were taken at approximately half the baseline collective target time, where no cells had reached the target. Velocities are shown with arrows and the speed is colour coded. (a). Rapid co-attraction response 1/(CoA rate) = 0.008. (b). Baseline conditions, where 1/(CoA rate) = 2. (c). Rapid response with 1/(CoA rate) = 0.008. Angle made with the vertical axes by leading, centre leading, centre, and centre trailing and trailing. As the data did not appear normally distributed a Wilcoxon signed rank test with continuity correction was applied to the leading and trailing data with a p-value<0.001. (d). Rapid response with 1/(CoA rate) = 0.008. Speed of cells partitioned by leading, centre leading, centre, centre trailing and trailing. As the data did not appear normally distributed a Wilcoxon signed rank test with continuity correction was applied to the leading and trailing data with p-value<0.001. (e). Baseline parameters. Angle made with the vertical axes by leading, centre leading, centre, and centre trailing and trailing. The same statistical test used in (c) and (d) indicated no significant difference between leading and trailing populations at baseline. (f). Baseline parameters. Speed of cells partitioned by leading, centre leading, centre, centre trailing and trailing. Wilcoxon signed rank test showed no significant difference between leading and trailing populations at baseline.
(TIFF)

Table S1 **List of parameters used in the discrete element model and their values.** Values were approximated from either experimental data, or through comparison of emergent behaviour between model and experiment. Where parameters have been chosen from sensitivity analysis, their error bounds are shown. ND represents a scalar parameter.
(DOCX)

Text S1 **Further details of the equations of motion, the analysis and supplementary results.**
(DOCX)

Video S1 *A*. Movie showing the four cases tested. Details from left to right: Random movement for the case **−CIL,−CoA.** *B*. Random movement similar to diffusion in the absence of co-attraction. *C*. High density meandering cluster, showcasing the inhibition of directional migration when CIL is inhibited. *D*. Baseline simulation, showing the mutual inclusion of CIL and co-attraction lead to directional migration.
(AVI)

Video S2 *A*. Movie showing random variation in directional group persistence time, attributed by group reorientation. *B*. Constant co-attraction promotes a leading trailing decomposition amongst simulated cells during directional migration.
(AVI)

Video S3
**Movie showing that a single cell cannot respond to co-attraction at a distance of** 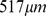 **, when placed in proximity to a group of 25 simulated cells.**
(AVI)

Video S4
**Movie showing that co-attraction acts a distance of** 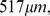 **, when two groups contain 25 simulated cells.**
(AVI)
