# Supplementary figures and images for: Directional Collective Cell Migration Emerges as a Property of Cell Interactions
Source: PLoS One. 2014 Sep 2;9(9):e104969. doi: 10.1371/journal.pone.0104969 (PMC4152153; doi:10.1371/journal.pone.0104969)

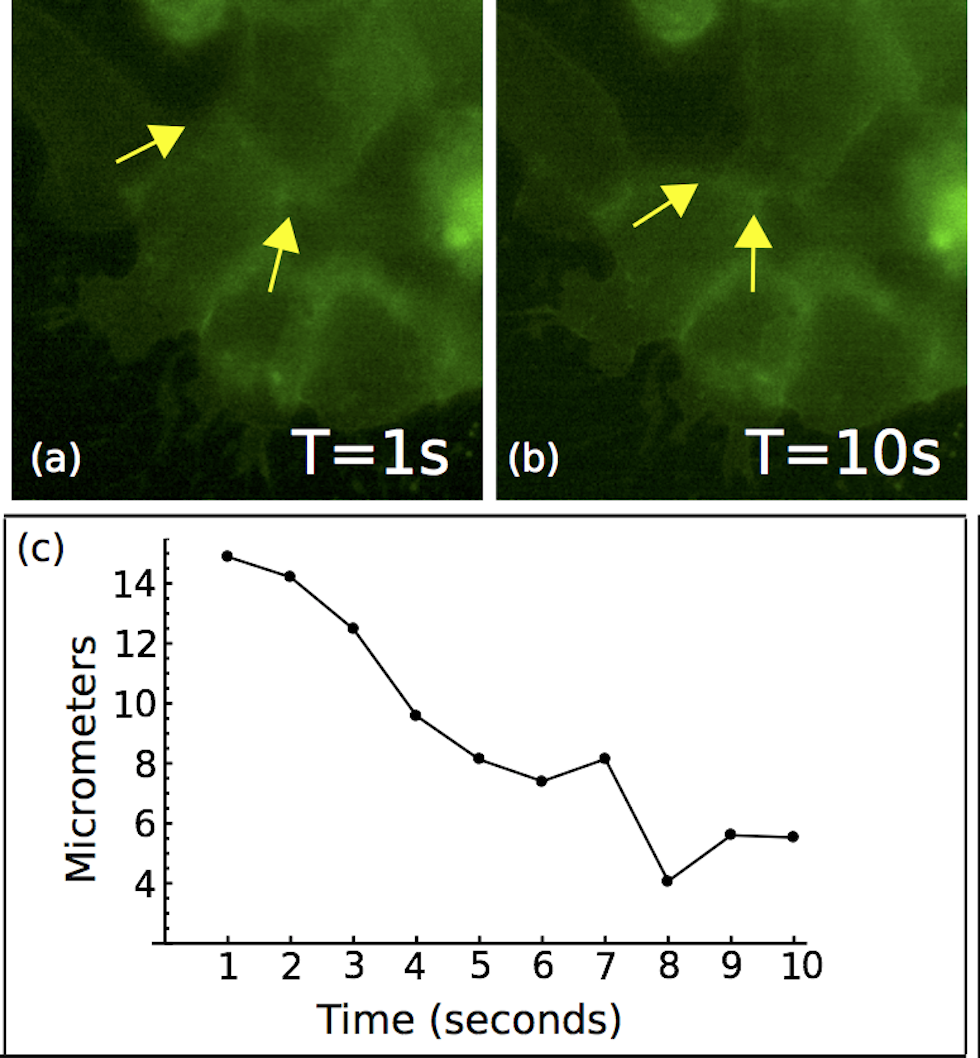

Supplement: Figure S1 — Contact time. (a). Frame from an in vitro experiment showing the contact area of two cells. (b). Frame after 10 seconds have elapsed from the time of frame in (a). (c). Length of contact area cross section, recorded over 10 seconds, with data from the experiment shown in (a) and (b). (TIFF) [file pone.0104969.s001.tiff]

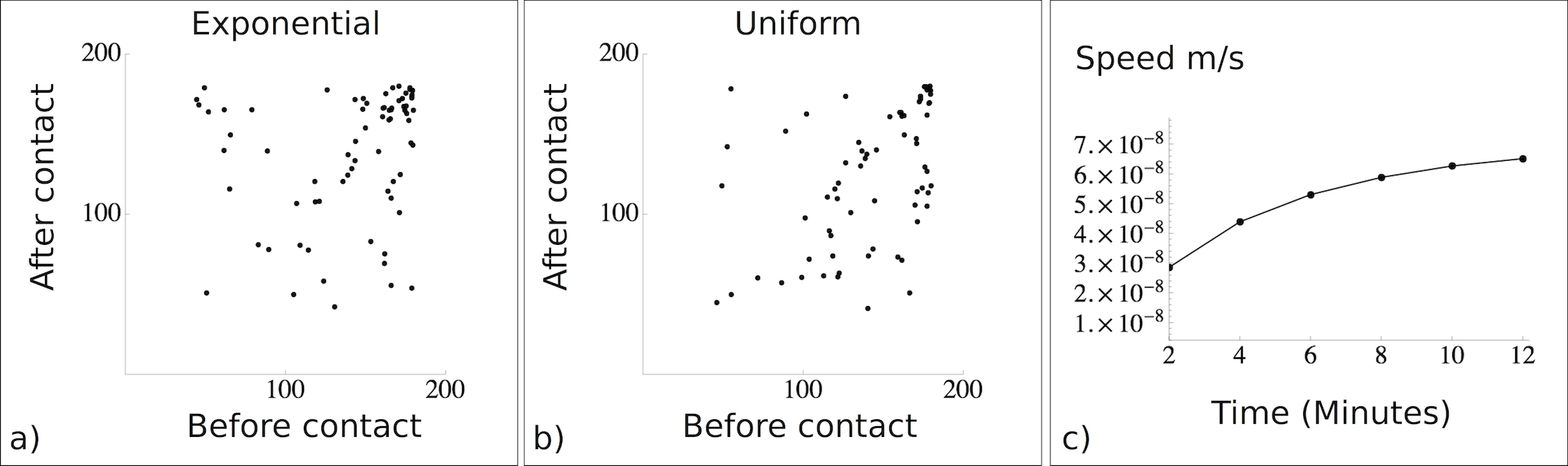

Supplement: Figure S2 — Testing repolarisation. (a–b). Repolarisation plots. (a). Exponential distribution with mean 18 degrees from normal vector connecting the cell's centre of mass. (b). Uniform distribution between and . (c) Speed after contact for the parameter value . (TIFF) [file pone.0104969.s002.tiff]

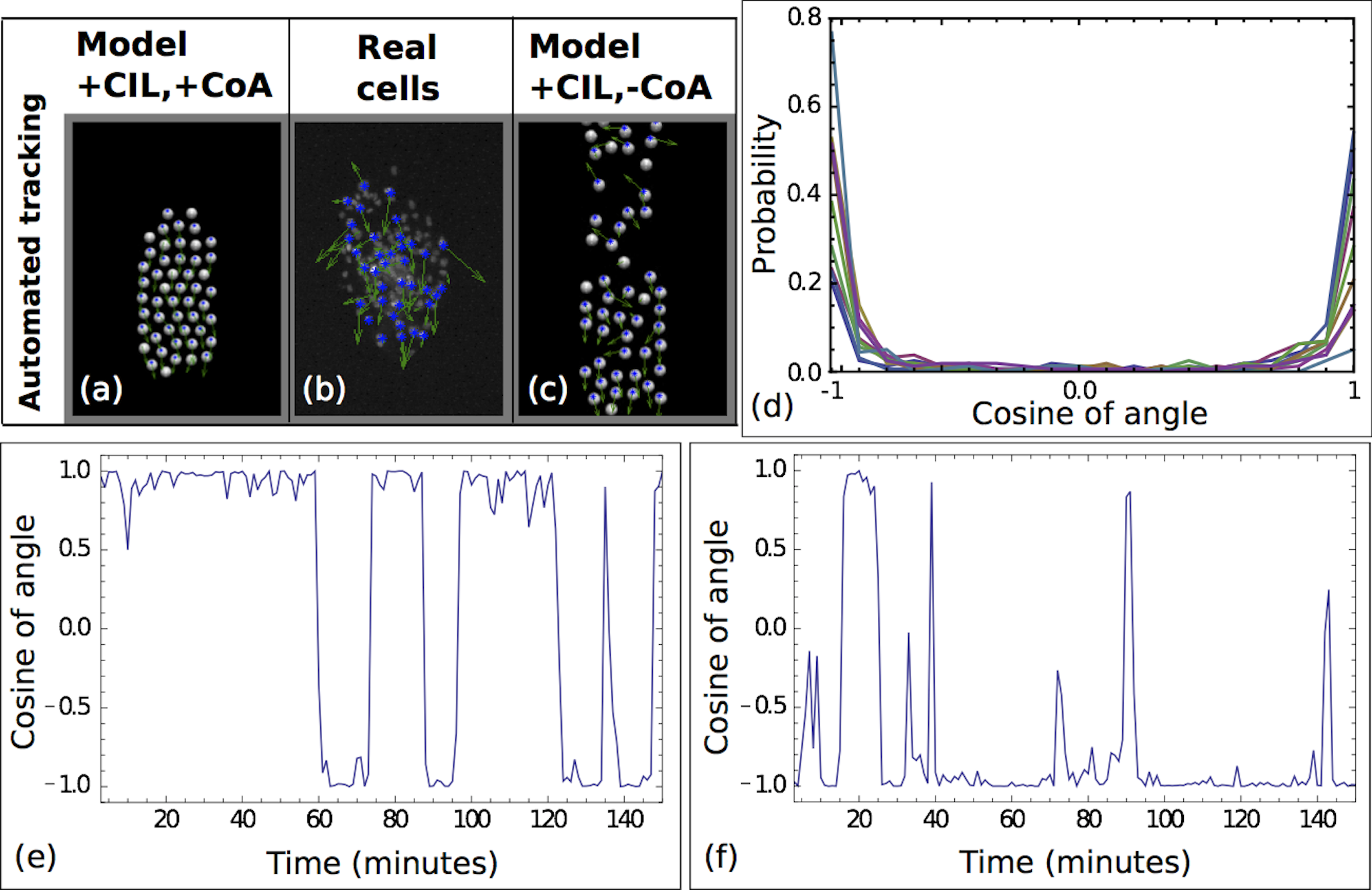

Supplement: Figure S3 — Tracking cell motion. (a). Time frame from the DIDSON tracking software for the model case (+CIL,+CoA). (b). Time frame from the DIDSON tracking software for a control group of NC cells plated on a strip of fibronectin. (c). Time frame from the DIDSON tracking software for the model case (+CIL−CoA). (d). Directional migration for the baseline case, showing the cosine of the angle made with the vertical axis for the average group direction. Average distribution of direction over the whole simulation, 10 independent simulations shown in different colours. (e). Time series of a single simulation, showing that persistence of direction can last for up to an hour in length and switches in direction can take place in a few minutes. (f). Time series for a single simulation, showing that a group can continually move in one direction for two hours subject to a few reorientations. (TIFF) [file pone.0104969.s003.tiff]

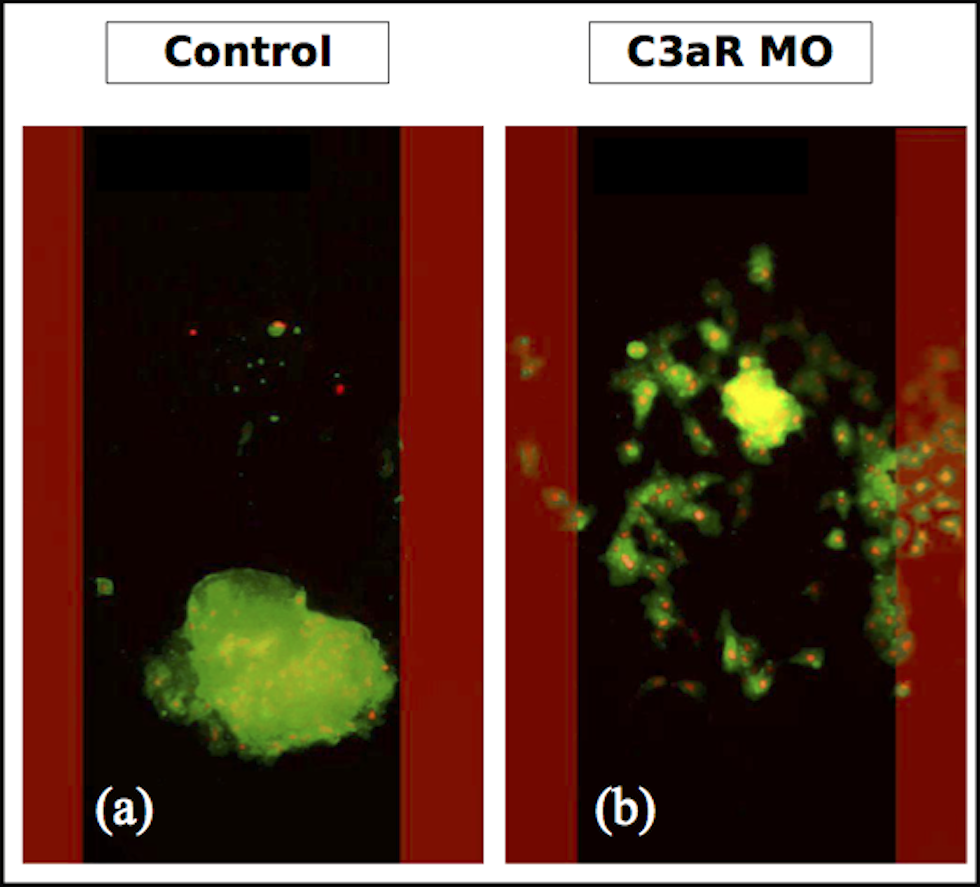

Supplement: Figure S4 — Co-attraction facilitates stream guidance. NC cultured on corridor of fibronectin (black area), flanked by non-permissive substrate (red area). (a) Control NC. (b) C3aR deficient NC, here cells are able to cross into the restricted region. (TIFF) [file pone.0104969.s004.tiff]

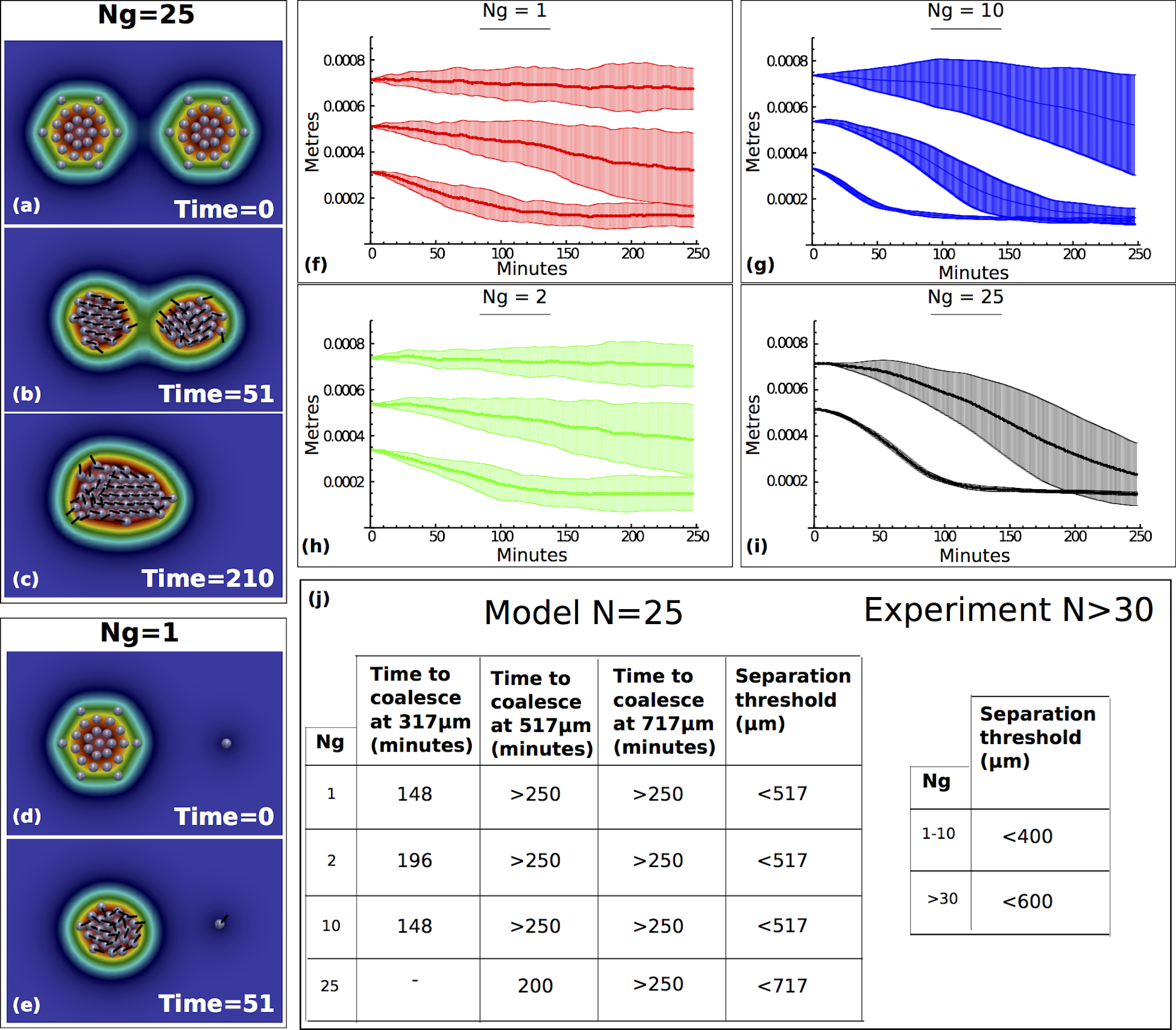

Supplement: Figure S5 — Co-attraction between two different sized groups. (a). , , initial condition, where the centre of mass separation was . (b). At a time of 51 minutes into the simulation, the groups begin the join. (c). At a time of 210 minutes, the two groups have responded to co-attraction and collectively migrate in a random direction. (d). Initial condition for the case , . (e). At time 51, in contrast to the simulation shown in (B), the single cell is disjoint from the larger group. (f). Time series showing the centre of mass separation for the three distances analysed, (see initial condition at time zero). At a distance of the single cell can migrate towards the reference group. (g). Same plot as shown in (f), for the condition , . (h). Same plot as shown in (f), for the condition , . (i). Same plot as shown in (f), for the condition , . (j). Table showing the results of the model and experiment. Time at which the groups have joined and the threshold at which groups can respond to co-attraction for all cases analysed. (TIFF) [file pone.0104969.s005.tiff]

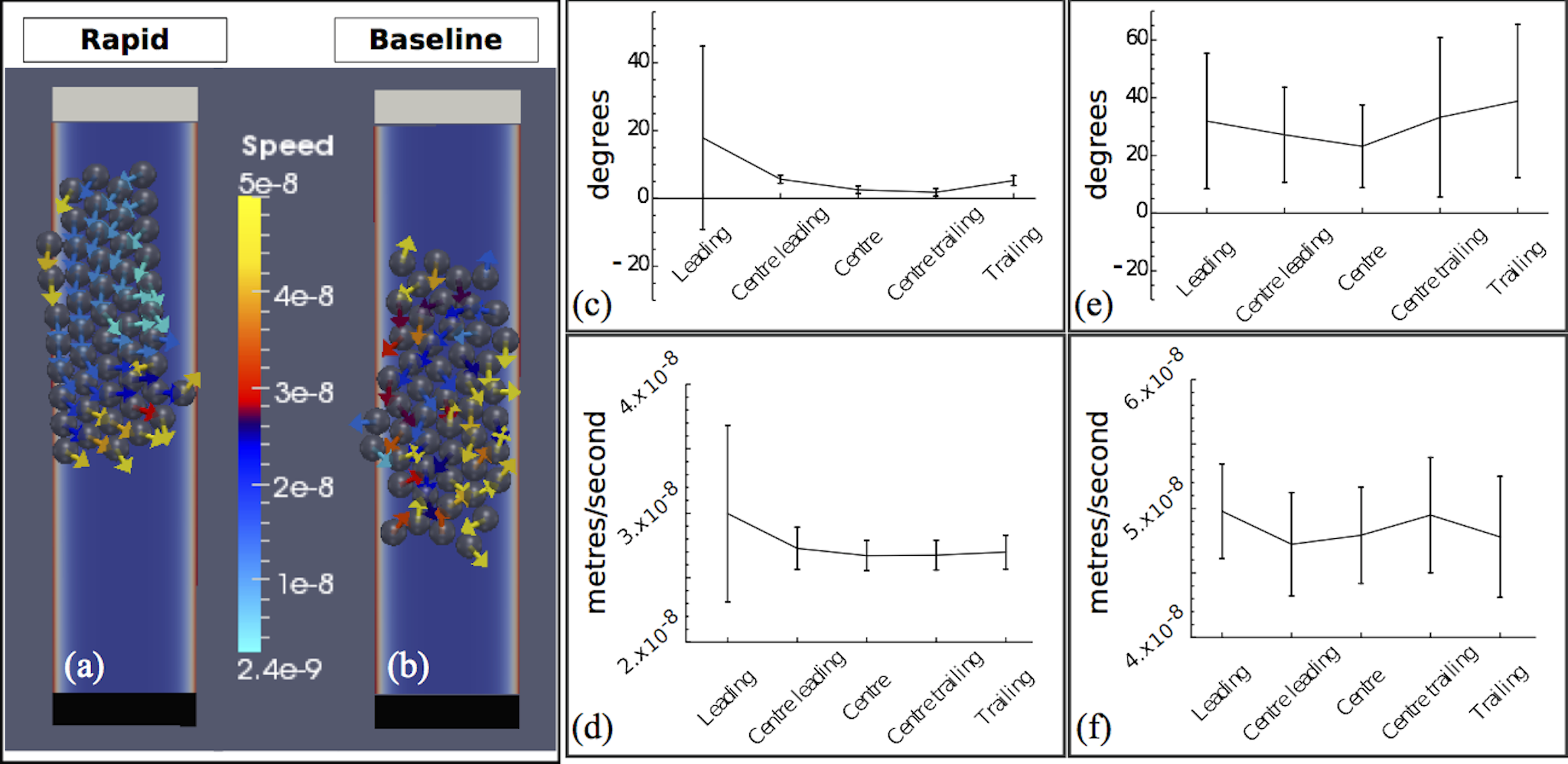

Supplement: Figure S6 — Leading and trailing cells. Images were taken at approximately half the baseline collective target time, where no cells had reached the target. Velocities are shown with arrows and the speed is colour coded. (a). Rapid co-attraction response 1/(CoA rate) = 0.008. (b). Baseline conditions, where 1/(CoA rate) = 2. (c). Rapid response with 1/(CoA rate) = 0.008. Angle made with the vertical axes by leading, centre leading, centre, and centre trailing and trailing. As the data did not appear normally distributed a Wilcoxon signed rank test with continuity correction was applied to the leading and trailing data with a p-value<0.001. (d). Rapid response with 1/(CoA rate) = 0.008. Speed of cells partitioned by leading, centre leading, centre, centre trailing and trailing. As the data did not appear normally distributed a Wilcoxon signed rank test with continuity correction was applied to the leading and trailing data with p-value<0.001. (e). Baseline parameters. Angle made with the vertical axes by leading, centre leading, centre, and centre trailing and trailing. The same statistical test used in (c) and (d) indicated no significant difference between leading and trailing populations at baseline. (f). Baseline parameters. Speed of cells partitioned by leading, centre leading, centre, centre trailing and trailing. Wilcoxon signed rank test showed no significant difference between leading and trailing populations at baseline. (TIFF) [file pone.0104969.s006.tiff]
