# Supplementary material for: Directional Collective Cell Migration Emerges as a Property of Cell Interactions
Source: PLoS One. 2014 Sep 2;9(9):e104969. doi: 10.1371/journal.pone.0104969 (PMC4152153; doi:10.1371/journal.pone.0104969)
Supplement: Table S1 — List of parameters used in the discrete element model and their values. Values were approximated from either experimental data, or through comparison of emergent behaviour between model and experiment. Where parameters have been chosen from sensitivity analysis, their error bounds are shown. ND represents a scalar parameter. (DOCX) [file pone.0104969.s007.docx]

| **Parameter** | **Value** | **Unit** | **Parameterisation** |
| --- | --- | --- | --- |
| Cell Mass $(m)$ | 1.0 e-10 | $kg$ | Here we approximate the density of a cell with three times the density of water and assume a radius of $20\mu m$ |
| Intrinsic speed $\left\vert v\right\vert$ | 5.0 e-8 | $m/s$  | Measured previously (SI Text reference [8]). |
| Domain Width $(W)$ | 217 e-6 | $m$ | Approximated from experimental data. |
| Domain Height $(H)$ | 850 e-6 | $m$ | Approximated from experimental data. |
| Chemotaxis Clock  CoA rate | 0.5 | $1/s$ | Chosen from sensitivity analysis: $0.003<$ 1/(CoA rate) < 20 |
| Boundary Clock $I_{iB}(t)$  rate | 0.1 | $1/s$ | Chosen from sensitivity analysis: 1/($I_{iB}(t)$ rate)<100 |
| Diffusion Length | 0.00011 | $1/m$ | Curve fit of stable gradient (SI Text reference [6]). |
| Constant of proportionality $(\beta)$ | 1 | $(m/s)^{2}$ | Models sensitivity to changes in co-attractant concentration. |
| Stiffness $(K)$ | 0.112 | $N/m^{3/2}$ | Calculated from material parameters; Assuming incompressibility and Poisson’s ratio of 0.5 and a Young’s modulus of 40 Pa (SI Text reference [2]). |
| Restitution | 2.199 | ND | Approximated from (SI Text reference [3]). |
| Time step $(\Delta t)$ | 1.0 e-3 | $s$ | Calculated from the mass and normal stiffness. |
| Number Of Cells $(N)$ | 50 | ND | Approximate number of cells in an experiment. |
| Contact adaption coefficient $(\alpha_{c})$ | 0.003 | $Ns/m$ | Experimental fit to data see figure S2g-i. |
| Migratory adaption coefficient $(\alpha_{p})$ | 1 | $1/s$ | Flexible parameter that can be adjusted to fit experimental data. |
| Rotational Turning Clock $\text{RT rate}$ | 0.003 | $1/s$ | Chosen from sensitivity analysis: 3<1/(RT rate) |
| Cell Radius $(r)$ | 20 e-6 | $m$ | Inspection of experimental data. |
| Coherence $(\left\langle V\right\rangle)$  | 0.5568 | ND | Control experimental value compared with the discrete element model under conditions with and without co-attraction in the model. |
