# Supplementary material for: Directional Collective Cell Migration Emerges as a Property of Cell Interactions
Source: PLoS One. 2014 Sep 2;9(9):e104969. doi: 10.1371/journal.pone.0104969 (PMC4152153; doi:10.1371/journal.pone.0104969)
Supplement: Text S1 — Further details of the equations of motion, the analysis and supplementary results. (DOCX) [file pone.0104969.s008.docx]

Directional collective cell migration emerges as a property of cell interactions

**Supplementary Material**

**Mae L. WOODS^1,2,4^, Carlos CARMONA-FONTAINE^2^, Chris P. BARNES^2^, Iain D. COUZIN^3^, Roberto MAYOR^2^ and Karen M. PAGE^1,4^**

^1^Centre of Maths Physics in the Life Sciences and Experimental Biology, University College London, London, England

^2^Department of Cell and Developmental Biology, University College London, London, England

^3^ Department of Ecology and Evolutionary Biology, Princeton University, Princeton, New Jersey, United States of America.

^4^ Department of Mathematics, University College London, London, England

**I. EQUATIONS OF MOTION**

In this study, each simulated cell is assigned identical update rules and variation observed is dependent on the initial conditions, stochastic repolarisation and the dynamical system.

**A. Self-propulsion**

The simulated cell speed parameter is assigned the default speed of a single NC cell. Variation in speed is accounted for by the relaxation to the average speed $|v|$, such that

$\bar{F}_{i}^{p}=\alpha_{p}m(\left|v\right|\hat{v}_{i}(t)-\bar{v}_{i}(t)).$ (1)

The self-propulsion force $\bar{F}_{i}^{p}$acts along the vector corresponding to the polarity of the simulated cell, where $\hat{v}_{i}(t)$ is a unit vector in the direction of polarity, $\alpha_{p}$ controls relaxation to the average speed, $m$ is the mass and $\bar{v}_{i}(t)$ is the velocity of the simulated cell at time step $t$.

**B. Attractive force**

The attractive force is proportional to the gradient of the chemoattractant spatial density at time $t$, evaluated at the end of the simulated cell’s ray of polarity:

(2)

Constructed in this way, the force generates a gradual increase and decrease in the force applied due to co-attraction. $\beta$ is a constant of proportionality and represents sensitivity to changes in co-attractant concentration and is the C3a concentration profile.

(3)

This concentration profile is the sum of those generated by each of the simulated cells, assumed to have reached steady state. For baseline conditions, $D=\ln(2)/0.00011m^{-1}$.

**C. Rotational Turning**

Periodically, simulated cells adopt a new polarity. This represents the sporadic re-orientation observed from isolated NC cells in biological experiments. The change occurs instantaneously via the application of an impulse.

$\bar{F}_{i}^{\omega}=m(\bar{v}_{\omega}(t)-\bar{v}_{i}(t))/\Delta t,$ (4)

where $\bar{v}_{\omega}(t)$ is the current velocity $\bar{v}_{i}(t)$ rotated by an angle $\omega$, and we use an effective force term.

**D. Contact Theory**

To implement normal contact forces, contact theory is applied. In this context, the scalar particle overlap is modelled as a function of a pressure distribution proposed by Hertz [1] such that

$\delta=\frac{\pi ap_{0}}{2E},$ (5)

where $\delta$ is the scalar particle overlap, $p_{0}$ is the maximum pressure, $a$ is the radius of the contact region and $E$ is the effective Young’s modulus.

$1/E=(1-\sigma_{i}^{2})/E_{i}+(1-\sigma_{j}^{2})/E_{j}$ (6)

$a=\frac{\pi p_{0}R}{2E}.$ (7)

Here, $E_{i}$ is the Young’s modulus for simulated cell $i$, and $\sigma_{i}$ is it’s Poisson ratio, $1/R=1/r_{i}+1/r_{j}$ is the effective radius and we assume identical material properties amongst simulated cells. By rearranging the above (5)(7), a contact force with a time dependent coefficient which is linear in the scalar particle overlap $k_{N}\delta,$ where $k_{N}=4/3Ea,$ can be defined with a constant coefficient and nonlinear function of the scalar particle overlap $K\delta^{3/2}$

$k_{N}\delta=\frac{4}{3}Ea\frac{a^{2}}{R}=\frac{4}{3}E\sqrt{R}\left(\frac{a^{2}}{R}\right)^{\frac{3}{2}}=K\delta^{\frac{3}{2}},$ (8)

where$K=4/3\sqrt{R}.$ From the literature $E_{i}=40Pa$ and we assume a homogenous incompressibility with $\sigma=0.5$ [2]. By substitution of these terms into (8), we obtain the value for the stiffness coefficient [3],

$K=8/9(40)\sqrt{0.00001}\cong0.112N/m^{\frac{3}{2}}.$ (9)

**E. Contact Force**

During contact between two simulated cells, standard normal contact forces are applied and a repolarisation force is initialized at the free edge opposite to the point of contact. Here the simulated cells are assumed to resist any torsional forces induced by tangential contact. We adopt the approach of Marshall [3] and treat the normal force as the sum of elastic deformation and energy loss terms, such that

$\bar{F}_{ik}^{C}+\bar{F}_{ik}^{Cd}=\left(K\delta_{ik}^{3/2}-\eta_{N}\bar{v}_{ikR}\cdot\hat{n}_{ik}\right)\hat{n}_{ik},$ (10)

where $K$ is the effective stiffness and can be expressed in terms of the effective Young’s modulus and contact region. $\bar{F}_{ik}^{C},$ $\bar{F}_{ik}^{Cd}$and $\delta_{ik}$ are the contact force, contact damping and scalar particle overlap and are defined in the main text. The contact damping is modelled with the relative velocity, $\bar{v}_{ikR}=\bar{v}_{i}-\bar{v}_{k},$ the normalized vector $\hat{n}_{ik}=\bar{u}_{i}-\bar{u}_{k}/|\bar{u}_{i}-\bar{u}_{k}|$ connecting the centroids of the two simulated cells in contact and the coefficient of restitution $\eta_{N}$. The coefficient of restitution is a function of the elastic stiffness and mass, scaled by a coefficient of friction. The repolarisation force $\bar{F}_{ik}^{R_{CIL}}$ is included to account for the biological intracellular processes that occur during CIL and is not present in standard discrete element models.

$\bar{F}_{ik}^{R_{CIL}}=\alpha_{c}\left(\left|v\right|\hat{n}_{ik}^{w}(t)-\bar{v}_{i}(t)\right),$ (11)

where $\left|v\right|$ is the speed parameter, $\hat{n}_{ik}^{w}$ is $\hat{n}_{ik}$ rotated by $w$ degrees to account for random repolarisation at the free edge and $w$ is randomly chosen for each simulated cell from a uniform distribution between $\pm90^{o}$. This analysis could be extended to model migration over or under the contacted simulated cell during CIL by developing an analysis in three dimensions, however the two-dimensional case is sufficient to generate a range of predictions on the role of CIL and co-attraction.

**F. Internal clocks**

The coefficient $Q$, defines the application of the self propulsion, attractive and rotational turning terms, such that,

Here $C$ is the set of neighbours in contact with the cell of interest. The coefficients $a_{i}(t),\omega_{i}(t)$ and $m_{i}(t)$ determine mutually exclusive application of the forces $\bar{F}_{i}^{a},\bar{F}_{i}^{\omega}$ and $\bar{F}_{i}^{p}$ when a cell is not in contact. This is controlled through the internal clocks governing co-attraction and rotational turning, $I_{iCoA}(t),$ a periodic step function and $I_{iRT}(t),$ a periodic impulse where both clocks alternate between zero and one. These internal clocks, for each simulated cell $i$ are randomly initialised, with times $t_{ai}$ and $t_{di}$ respectively and are defined explicitly with the following equations.

$I_{iCoA}(t)=H\left(\sin(\text{k}(t+t_{ai}))-\sin(\xi)\right)$ (12)

$I_{iRT}(t)=\sum_{z=0}^{\infty} \delta_{t(t-zM_{RT}-t_{di})}$ (13)

Here, $H(t)$ is the Heaviside function, $t_{ai}$ determines the initial value, $\text{k}$ and $\xi$ are real numbers that determine the co-attraction response rate. The function$I_{iRT}(t)$ is defined as an impulse where $\delta_{t(t-zM_{RT}-t_{di})}$ is Kronecker’s delta with period $M_{RT}$initialised at $t_{di}$.

Defining the clocks in this way permits the explicit definition of the coefficients, where $a(t)=I_{iCoA}(t),$ $\omega(t)=(1-I_{iCoA}(t))I_{iRT}(t)$ and $m(t)=(1-I_{iCoA}(t))(1-I_{iRT}(t))$.

When a simulated cell is not in contact with another simulated cell, the default force applied is self-propulsion. In the case $I_{iCoA}(t)=1,$ co-attraction is invoked. If $I_{iCoA}(t)=0$ and $I_{iRT}(t)=1,$ polarity will be lost and a new polarity will be adopted through angular deviation.

**G. Lateral borders**

To implement a response to negative signaling at the lateral borders, in addition to $I_{iCoA}(t)$ and $I_{iRT}(t),$ each simulated cell is equipped with a clock $I_{iB}(t)$ to periodically respond to a negative signal.

$I_{iB}(t)=H\left(\sin(k_{B}(t+t_{Bai}))-\sin(\xi_{B})\right),$ (14)

where as above, $H(t)$ is the Heaviside function, $t_{Bai}$ determines the initial value, $k_{B}$ is an integer and $\xi_{B}$ is a real number that determines the boundary response rate. The negative signal is modeled as a source at the boundary, with half maximum value at one half of the cells radius into the domain. In the simulations, the magnitude of the source was taken to be 10.5. When $I_{iB}(t)=1,$ a boundary force $\bar{F}_{i}^{b}$ is applied to cell $i$, such that

$\bar{F}_{i}^{b}=m\left|\nabla Q\right|\left(\left|v\right|\hat{q}_{i}(t)-\bar{v}_{i}(t)\right).$ (15)

Here, $\hat{q}_{i}(t)$ is a unit vector in the direction opposite to the gradient in the negative signal $Q$.

**H. Time discretization**

To simulate the results, the time step was taken to be equal to $0.001s.$ This is due in part to the critical time step and to data analysis. In order for the simulations to be stable, the time step should be a fraction of the critical timestep $\Delta t_{c},$ for the discrete element method without the repolarisation force [4]. This critical time step is given below.

$\Delta t_{c}\approx2\sqrt{\frac{m}{k}}$ (16)

$\Delta t<\Delta t_{c}$ (17)

For the simulations $\Delta t$ is taken to be $1.0\times10^{-3}$seconds. To interoperate this value in the context of real cells, the contact area time dependence during CIL was measured. Results were obtained from a movie with a resolution that enabled the contact area to be detected (figure S1a-b). By recording the length between the contact edge points over time, we infer that the contact area changes on a scale less than $1s$ (figure S1c). This microscopic timescale results in the requirement of high performance computing because to generate physiological predictions on the long range properties of collective migration two conditions must be satisfied. The time step must be of a resolution such that it can physically describe the microscopic interactions and the simulation length must be on the order of hours so that the emergent properties can stabilise.

**II. QUANTIFYING EFFICIENCY**

To quantify the dependence of migration on CIL and co-attraction, a systematic technique to measure motion was adopted. This allowed collective migration to be defined by two global measures, a coherence parameter and collective target time. The coherence parameter, previously shown to be an order parameter for similar systems [5] and the biologically motivated collective target time were used and compared to test the hypothesis that CIL and co-attraction are sufficient for directional migration. The coherence parameter $\left\langle V\right\rangle$ was the sum of the normalized velocities averaged over the number of cells and coherence time $t_{k},$ where coherence was measured 30 minutes after initialization and for the duration of $t_{k}=1$ hour.

$\left\langle V\right\rangle=\left\langle\frac{1}{N}\left|\sum_{i=1}^{N} \frac{\bar{v}_{i}(t_{k})}{\left|\bar{v}_{i}(t_{k})\right|}\right|\right\rangle_{t_{k}}.$ (18)

The collective target time $t_{T}$ was the time taken for 80% of the population to reach the target boundary.

**III. AUTOMATED TRACKING**

To determine if the coherence assessment is an effective measure of collective migration in the NC, automated software was applied to a control *in vitro* experiment and model baseline scenario. A velocity field was generated from the software and the frame rate was taken to be 3 minutes in both the experimental and simulated data (figure S3a-c).

**IV. EMERGENT PROPERTIES**

**A. Collective flight time**

There was variation in the collective target time between individual runs under baseline conditions. This presents an uncertainty in the length of time that the group remains travelling in one direction, which we define as a collective flight time. In some cases the group is able to self organize such that it travels continually to the target domain, however in other cases the group experiences rotation and re-orientates to travel back up the channel. This variation in the collective target time for baseline conditions is negligible when compared to the collective target time for cases in which CIL or co-attraction are inhibited. This uncertainty in collective flight time leads to the hypothesis that directional migration could potentially promote a flexible system that is ready to adapt to perturbations, see Video S2A.

To test perturbation of the collective, the influence of the dorsal boundary condition was investigated. Simulated cells were initialised at the longitudinal mid point of the corridor and the average direction of the group was recorded. Groups that migrated in the direction of the target, such that the dot product of the average direction with the negative vertical axis was positive, were recorded as a positive result. Groups that migrated towards the dorsal region of the domain were recorded as a negative result. The time course of this value for 10 independent simulations is shown in (figure S3d). The persistence of direction can be seen in (figure S3e-f). It is proposed that although external signals are not required to generate directional migration, proximal chemoattractants may facilitate guidance towards a target.

**V. MODEL PREDICTIONS**

**A. Co-attraction facilitates stream guidance**

The NC migrate in streams of cells because there are negative signals at the border of each stream that prohibit the migration of NC cells into these areas. However, by reducing co-attraction using antisense morpholinos against C3aR, it can be verified experimentally that co-attraction is necessary to prevent cells moving outside the lateral boundaries *in vitro* (figure S4a-b). This represents a new function of co-attraction and a mechanism to maintain stream migration. Figure S4a shows the control migration of the NC. When co-attraction is inhibited, (figure S4b), single cells fail to interpret the negative signals and disperse into the surrounding tissue.

**B. Co-attraction of clusters**

It has previously been shown that co-attraction regulates the attraction of groups that have been separated [6]. This result is reproducible with the discrete element model (figure S5). In contrast to previous attraction and repulsion models, [6], the discrete element model is parameterised with experimental data at the single cell level. Experimental data shows, that the distance over which co-attraction can act is dependent on the number of cells within each group (figure S5j). The advantage of experimental parameterisation, is that it allows this experimental data to be compared with the emergent properties of the model. It is possible to determine the distance over which co-attraction can act for different sized groups in the model. To obtain a prediction on the distance, we defined a reference group of $N=25$ simulated cells and subgroup $Ng$ (figure S5a-e). We tested values of $Ng=1,2,10$ and 25 simulated cells. To perform the analysis, the distance between the centre of mass was recorded throughout the time series for time intervals of 3 minutes and ten independent simulations, (figure S5f-i). Distances of $317\mu m,$ $517\mu m$ and $717\mu m$ were tested. The distance $317\mu m$ was omitted in the case $Ng=25$because the groups were able to co-attract at a distance of $517\mu m.$ To standardise the point at which groups were considered to have joined, the time point at which the distance between the two centres of mass was within $200\mu m$ was recorded. In addition, the difference between the distance at consecutive time frames was required to be less than $2\mu m$ (figure S5j). The results of this analysis predicted that groups of 25 simulated cells can co-attract within a distance less than $717\mu m$ and groups of 1,2 and 10 cells require a shorter distance and co-attract at distances less than $517\mu m$. This analysis was compared with experimental data. For a reference group of $N=30$ cells, groups of 1-10 cells can co-attract at distances less than $400\mu m$ and groups of 30 cells can co-attract at distances less than $600\mu m$ (figure S5j).

**C. Leading and trailing decomposition**

A difference between leading and trailing populations occurred in the angular deviation from the vertical axis and in the speed. The difference was recorded by dividing the group of simulated cells into five partitions of size $N=10$ cells along the vertical axis and labeling them leading, centre leading, centre, centre trailing and trailing. For values of 1/(CoA rate)<0.1, leader cells adopted a speed close to the default speed parameter $\left|v\right|$ and deviated from the vertical corridor axes, whereas trailing cells adopted smaller speeds and remained aligned with the corridor axes (figure S6a). For a co-attraction response at baseline, leading and trailing partitions apparently disappear (figure S6b). Quantification of this difference shows that for rapid co-attraction, there are significant differences between the leading and trailing simulated cells, despite the single simulated cell behaviour being the same (figure S6c-d).

Stress heterogeneity has been observed in migratory epithelial cell types such as endothelial cells [7] and here we propose that a heterogeneous stress distribution could also arise in mesenchymal cells and is accounted for by co-attraction.

**Software**

Parameter sweeps were performed in parallel and coded with CUDA provided by NVIDIA. Data was processed in PARAVIEW, ImageJ, DIDSON tracking software, R and Mathematica.

**References**

1. K.L.Johnson, Contact Mechanics, (CambridgeUniversityPress,Cambridge) 1st pbk. (with corrections) Ed pp xi, 452 p.

2. J. Zhou, H.Y. Kim, J.H. Wang and L.A. Davidson, Macroscopic stiffening of embryonic tissues via microtubules, RhoGEF and the assembly of contractile bundles of actomyosin, Development, 137(16) (2010), pp. 2785–2794.

3. J.S. Marshall, Discrete-element modeling of particulate aerosol flows, J Comput Phys., 228(5) (2009), pp. 1541–1561.

4. P.A. Cundall and O.D.L. Strack, Discrete Numerical-Model for Granular Assemblies, Geotechnique, 29(1) (1979), pp. 47–65.

5. B. Szabo, G.J. Szollosi, B. Gonci, Zs. Juranyi, D. Selmeczi and T. Vicsek, Phase transition in the collective migration of tissue cells: experiment and model, Physical review. E, Statistical, nonlinear, and soft matter physics. 74(6 Pt 1) (2006)

6. C.Carmona-Fontaine, E.Theveneau, A.Tzekou, M.Tada, M.Woods, K.M.Page, M. Parsons, J.D. Lambris and R. Mayor Complement fragment C3a controls mutual cell attraction during collective cell migration, Developmental cell, 21(6) (2011), pp. 1026–1037.

7. D.T. Tambe, C.C. Hardin, T.E. Angelini, K. Rajendran, C.Y. Park, X. Serra- Picamal, E.H. Zhou, M.H. Zaman, J.P. Butler, D.A. Weitz, J.J. Fredberg and X. Trepat Collective guidance by cooperative intercellular forces, Nat. Materials, 10(6), (2011). pp. 469–475.

8. Carmona-Fontaine C, Matthews HK, Kuriyama S, Moreno M, Dunn GA, et al. (2008) Contact inhibition of locomotion in vivo controls neural crest directional migration. Nature 456: 957-961.
